# Lapiferin protects against H1N1 virus-induced pulmonary inflammation by negatively regulating NF-kB signaling

**DOI:** 10.1590/1414-431X20209183

**Published:** 2020-09-07

**Authors:** Lishu Pei, Xuejin Gao, Wen Liu, Xiao Feng, Zhongquan Zhao, Yanping Lai

**Affiliations:** 1 Department of Respiration, The Second Hospital of Tianjin Medical University, Tianjin, China; 2 Department of Respiration, Xiqing Hospital, Tianjin, China; 3 Department of Respiration, Peking University Third Hospital, Beijing, China

**Keywords:** Lapiferin, Pneumonia, Virus, NF-κB, Inflammatory factors

## Abstract

H1N1 virus-induced excessive inflammatory response contributes to severe disease and high mortality rates. There is currently no effective strategy against virus infection in lung. The present study evaluated the protective roles of a natural compound, lapiferin, in H1N1 virus-induced pulmonary inflammation in mice and in cultured human bronchial epithelial cells. Initially, Balb/C mice were grouped as Control, H1N1 infection (intranasally infected with 500 plaque-forming units of H1N1 virus), lapiferin (10 mg/kg), and H1N1+lapiferin (n=10/group). Lung histology, expression of inflammatory factors, and survival rates were assessed after 14 days of exposure. Administration of lapiferin significantly alleviated the virus-induced inflammatory infiltrate in lung tissues. Major pro-inflammatory cytokines, such as interleukin (IL)-1β, IL-6, and tumor necrosis factor (TNF)-α, were decreased at both mRNA and protein levels by lapiferin administration in the lung homogenate. Lapiferin also reduced inflammatory cell numbers in bronchoalveolar fluid. Mechanistically, lapiferin suppressed the transcriptional activity and protein expression of NF-κB p65, causing inhibition on NF-κB signaling. Pre-incubation of human bronchial epithelial cells with an NF-κB signaling specific activator, ceruletide, significantly blunted lapiferin-mediated inhibition of pro-inflammatory cytokines secretion in an air-liquid-interface cell culture experiment. Activation of NF-κB signaling also blunted lapiferin-ameliorated inflammatory infiltrate in lungs. These results suggested that lapiferin was a potent natural compound that served as a therapeutic agent for virus infection in the lung.

## Introduction

Infection caused by influenza viruses poses a considerable threat to human health around the world. Swine-origin influenza A (H1N1) virus first emerged in humans in April 2009 in Mexico (1). It then spread across 214 countries and resulted in 18,449 deaths by 2010 (2,3). Based on a recent report, a total of 12.8 million cases were reported in mainland China alone with 805 deaths, making up a 0.5% mortality rate (4). Among the primary causes of H1N1-induced deaths, acute pneumonia was the most predominant (5). After infection, an estimated 9-31% of the patients required admission to intensive care units (6,7).

To date, treatment strategies for severe pneumonia rely on different types of adjunctive therapies, including neuraminidase inhibitors (i.e., oseltamivir) and antiviral compounds such as ribavirin. However, these therapies are non-specific and antiviral therapy usually involves a delayed response or has low efficacy (8,9). Systemic corticosteroids are the preferred therapy for severe influenza infection (10), but this may increase the risk of mortality (11). Therefore, more effective treatment agents against virus-induced pneumonia should be investigated.

Traditional Chinese medicine (TCM) has an important role in the treatment of infectious diseases in China (12). Among the therapeutic TCMs, lapiferin is a novel agent that is a complex ester of sesquiterpene alcohols with aliphatic acid and does not possess ionophoric properties (13). Lapiferin is extracted from the roots of *F. lapidosa* and its structure and configuration have been established based on chemical transformation and analysis of spectral characteristics (14). The crystal data of lapiferin is as follows: C_22_H_34_O_6_, mol. wt.=394.49, crystal size 0.54×0.53×0.42 mm^3^. Recently, lapiferin has been shown to exert biological effects. For example, it protects against human colon cancer cell proliferation *via* interaction with type II estrogen-binding sites (15). Similarly, lapiferin induced cell apoptosis in breast cancer MCF-7 cells (16). Despite increasing evidence indicating the significance of lapiferin in cancer therapy, its role in human infectious diseases remains largely unknown.

NF-κB signaling pathway is one of the most important pathways that is activated under a pathogen challenge (17,18), and its modulation confers a therapeutic target in autoimmunity (19). The members of the nuclear factor kappa-light-chain-enhancer of activated B cell (NF-κB) family in mammalian cells include the proto-oncogene c-Rel, p50/p105 (NF-κB1), p65 (Rel A), p52/p100 (NF-κB2), and Rel B (19). These proteins share a conserved 300-amino acid region known as the Rel homology domain, which is responsible for DNA binding, dimerization, and nuclear translocation of NF-κB. The p65 subunit is a major component of NF-κB complexes and is responsible for transactivation. NF-κB heterodimeric p65-p50 and p65-c-Rel complexes are transcriptional activators. IκBα is an NF-κB inhibitor that could be phosphorylated and degraded when cells suffer a virus challenge (20). The inhibitory effect of IκB on NF-κB is exerted primarily through the interaction with p65. p65 shows a weak DNA-binding site, which could contribute directly to DNA binding in the NF-κB complex. It associates with chromatin at the NF-κB promoter region via association with DDX1 (20).

The present study aimed to evaluate the role of lapiferin in H1N1-induced pneumonia *in vitro* and *in vivo*.

## Material and Methods

### Ethical approval

Protocols for animal experiments were approved by the Ethical Committee at the Second Hospital of Tianjin Medical University (China).

### Virus, cells, agents, and animals

Influenza A/Beijing/501/2009 (H1N1) (BJ501) was obtained from the Beijing Institute of Microbiology and Epidemiology, State Key Laboratory of Pathogen and Biosecurity (China). Specific pathogen-free (SPF) Balb/C male mice, 4-6 weeks old, weighing 14-16 g, were purchased from the animal center at the Second Hospital of Tianjin Medical University. Mice were housed in a controlled atmosphere. They had free access to food pellets and tap water under standard conditions of humidity (50±10%), temperature (25±2°C), and a 12-h light/dark cycle. Human bronchial epithelial cells were purchased from the Cell Bank of Chinese Academy of Science (China). After adaptation for 1 week, mice were grouped (n=10/group) and received corresponding treatments as indicated. Lapiferin was purified and purchased from the small molecular library at the Chinese Academy of Science (China) and dissolved in PBS containing 0.1% DMSO. The dose for *in vivo* use of lapiferin was 10 mg/kg and 2 μg/mL for *in vitro* cell treatments. NF-κB specific activator ceruletide was purchased from Med Chem Express (USA) and used as a final dose of 1 μg/mL in cell culture or 2 mg/kg for animal treatments. The final doses of lapiferin and ceruletide were chosen based on a preliminary dose-dependent analyses of pro-inflammatory factors in cultured human bronchial epithelial cells (Supplementary Figure S1). In brief, to select the proper final doses, human bronchial epithelial cells were cultured with lapiferin or ceruletide at various doses for 24 h and then the cell supernatants were collected and subjected to ELISA detection of pro-inflammatory factors IL-1β and TNFα. The final dose was chosen based on maximal changes of pro-inflammatory factors with the lowest dose.

### Mouse model

**Figure 1:**
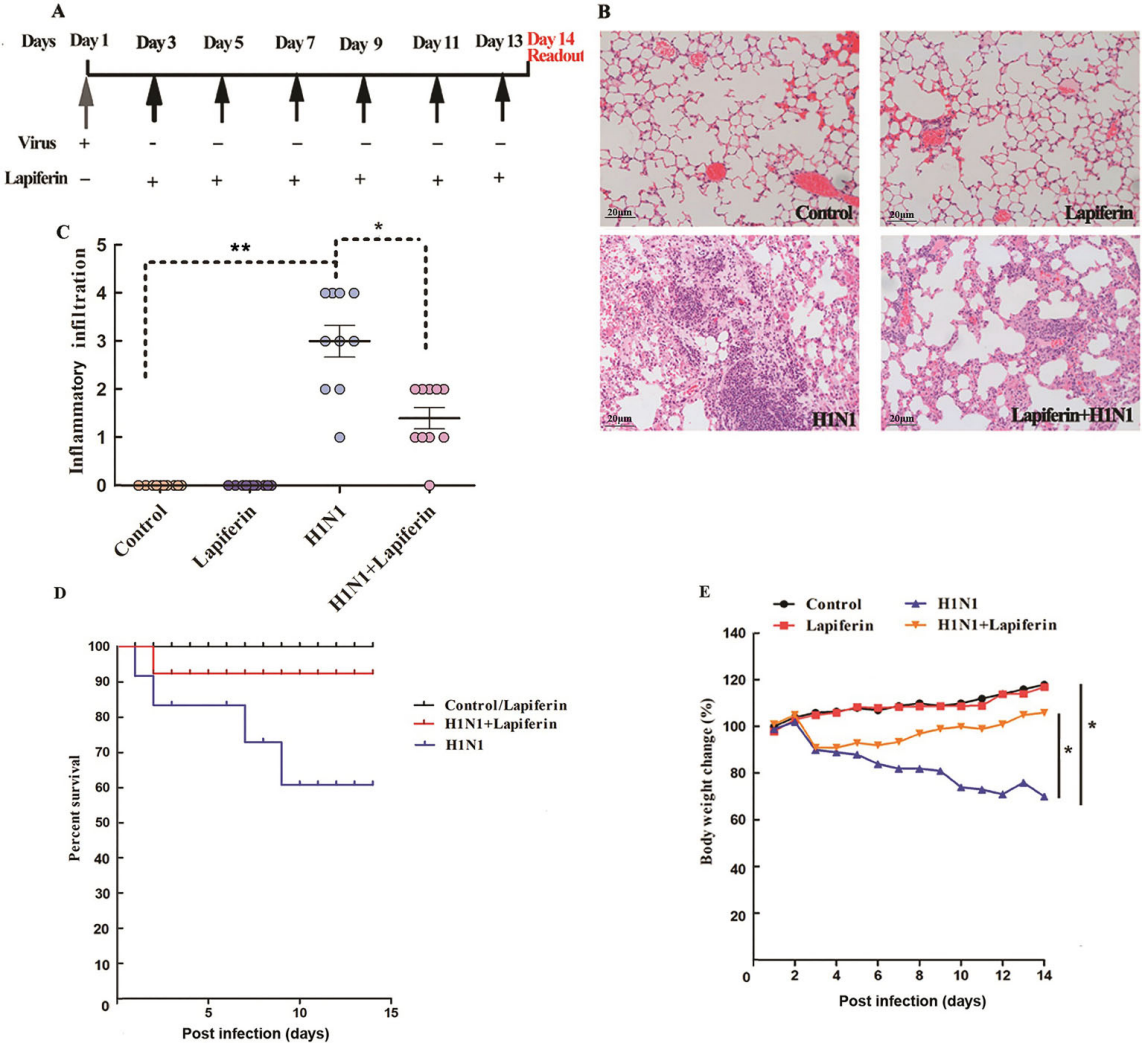
Lapiferin attenuated H1N1-induced mortality and histopathology in lungs. **A**, Experimental treatment protocols. **B**, Fourteen days after H1N1 infection, lung tissues were subjected to paraffin embedding and subsequent HE staining. Representative images from each group are shown (magnification: 400×, scale bar 20 μm). **C**, Semiquantitative analysis (scores) of inflammatory infiltrate in lungs of each group of mice. **D**, Mouse mortality from each group (n=10 per group). **E**, Body weight change from each group of mice. *P<0.05, **P<0.01 as indicated (ANOVA).

To establish a murine virus-induced pneumonia model, mice were intranasally infected with H1N1 virus (H1N1 group, n=15). Control mice were treated with PBS (control group, n=15). Virus-infected mice were given a total of 30 μL viral suspension/mouse (viral titer 500 plaque-forming units) under anesthesia with isoflurane in a Biosafety Level-2 (BSL-2) laboratory (4,21). One day after H1N1 infection (on day 3), mouse survival rate and body weight significantly dropped (Figure 1D and E). Therefore, lapiferin treatment was started on day 3. To mimic the common manner of drug delivery, lapiferin was injected intraperitoneally into H1N1-infected mice every other day (H1N1+lapiferin group, n=15). Mice treated with the same amount of lapiferin also formed the lapiferin group (n=15). Each group of mice was monitored for 14 days. Body weight and mouse survival in each group were monitored on a daily basis. The NF-κB activator ceruletide was intraperitoneally injected on day 2 and day 8 after H1N1 infection with or without lapiferin treatment (H1N1+ceruletide group and H1N1+ceruletide+lapiferin group, n=15/group). Before the use of ceruletide, a single injection was performed to evaluate its toxic effect (ceruletide group, n=15). Five mice were randomly selected from each group at 4 days post-infection (DPI) and subjected to viral load and pulmonary edema assessment as stated below. The remaining mice in each group were continuously monitored during the whole experimental periods. At the end of experiments, all mice were sacrificed by cervical dislocation and all efforts were made to minimize suffering. Humane endpoints were applied by chloral hydrate intraperitoneal injection when mice were sedentary, lacked active response upon stimulus, and ate less (4).

### Viral load determination in the lung tissues of mice

Lung tissues from five mice selected from each group were collected at 4 DPI. The titers of viruses in the lung tissue were determined by the cell culture infective dose 50% (CCID50) assay in Madin Darby canine kidney cells, as described previously (22).

### Acute pulmonary edema assessment

Assessment of acute pulmonary edema (wet-to-dry ratio) was performed at 4 DPI by calculating the right lung wet-to-dry ratio from the weight of wet lungs and the dry weight obtained after heating the tissues at 68°C for 24 h, as described previously (4).

### Measurement of immune cell numbers in bronchoalveolar (BAL) fluid

On the last day of the experiment, 1 h before mice were sacrificed, mice were intraperitoneally injected with pentobarbital. Lungs were then lavaged twice with 0.8 mL of lavage buffer (PBS containing 1% BSA). Cell counts in BAL fluid were measured and differentiated using a Sysmex XT-1800i automated hematology analyzer (SYSMEX, Japan).

### Air-liquid-interface (ALI) cell culture experiments

The ALI cell culture was performed in accordance with previous literature (19). The human bronchial epithelial cells were obtained from Lonza Bioscience (Germany; Product code 00195275S; Lot No. 0000332498). Cells were thawed and cultured in a T75 culture flask overnight in expansion medium (PneumaCult-Ex basal medium containing 10% PneumaCult-Ex supplements (Ybio, China), 1 μL/mL hydrocortisone). Cells were grown to a confluency of 80%. Subsequently, reagent pack subculture reagents (Lonza Bioscience) were used for trypsination. Cells were counted and transferred into a falcon tube. A total of 35,000 cells were added onto Transwell^®^ Permeable Support filters (6.5-mm insert, 0.4-μm polyester membrane, Costar, USA). Four days after addition of the cells onto the transwell filters, apical medium was removed and basal medium was switched to differentiation medium (Pneumacult-ALI basal medium containing 10% Pneumacult-ALI supplements, 1% Pneumacult-ALI maintenance supplements, 2 mL/mL 0.2% heparin sodium salt in PBS, 4 mL/mL hydrocortisone). ALI cells were grown for three weeks and differentiated into basal cells, ciliated cells, and mucus-producing cells. Medium was changed every second day. Apical mucus was removed as required by washing with PBS. Hydrocortisone was removed from the medium five days prior to treatment and H1N1 infection. Appropriate isotypes were used as controls. Readout was performed 48 h post-infection.

### Cytokine measurements

For cytokine measurements, lungs were removed and homogenized using a FastPrep-24 Sample Preparation System (MP Biomedicals, USA). Briefly, after dissection, lungs were perfused with PBS by pulmonary artery in order to remove intravascular blood. Then, lungs were rinsed in PBS twice and dried on a paper towel. Hank's salt solution, consisting of 1% BSA, 0.1% 0.5M EDTA, protease and RNase inhibitor, was used as homogenization buffer (23). The lung homogenates or supernatants in the cell culture were then subjected to enzyme-linked immunosorbent assay (ELISA). Briefly, mouse ELISA kits for IL-1β, IL-6, TNF-α, and fractalkine were purchased from the eBioscience (USA). Protocols for detection were in accordance with the manufacturers' instruction. The absorbance of each group for each indicator was determined at a wavelength of 450 nm with a microplate reader (Thermo Fisher, USA).

### Western blot

Lung tissues were lysed using RIPA lysis buffer (Beyotime Tech, China). After quantification using a BCA kit (Bio-Rad, USA), an equal amount of proteins was loaded to a 10% SDS-PAGE and then transferred to polyvinylidene fluoride membranes using a Trans-Blot Turbo blotting system (Bio-Rad). The membranes were blocked by 5% skim milk and incubated with primary antibodies overnight with the desired dilution concentration. After being washed three times in Tris-buffered saline containing 0.1% Tween 20 (TBS-T, pH 7.5), they were treated with corresponding secondary antibodies. Membranes were then washed three times with TBS-T and the protein bands were visualized using enhanced chemiluminescence (ECL, Thermo Fisher) solution and photographed using a ChemiDoc XRS Plus image analyzer (Bio-Rad). Primary antibody against NF-κB p65 was purchased from Abcam (China) and antibodies against IL-1β, IL-6, TNF-α, VCAM-1, and ICAM-1 were purchased from Cell Signaling Technology (USA). GAPDH was used as a loading control and its specific primary antibody was purchased from Santa Cruz Biotechnology (USA).

### Quantitative real-time polymerase chain reaction (qRT-PCR)

Total RNAs from lung tissues were extracted with Trizol reagent (Takara, Japan), according to the manufacturer's instruction. Total RNA (1 µg) was immediately reverse-transcribed into cDNAs with ReverTraAceqPCR RT Master Mix (Toyobo, Japan). qPCR was performed with Thunder bird SYBR qPCR Mix (Toyobo) and the ABI 7900 Thermal Cycler system. The stepwise protocols involved an initial incubation at 95°C for 1 min, followed by 35 cycles at 95°C for 10 s and 60°C for 30 s. Relative expression of target mRNA was determined using the 2^-ΔΔCT^ method. The *gapdh* gene was used as a reference gene. The primer sequences used for qRT-PCR are listed in Table 1. Experiments were repeated three times with each assay in triplicates.

**Table 1:** Primers used in this study.

Genes	Forward (5′-3′)	Reverse (5′-3′)
IL-1β	GAAATGCCACCTTTTGACAGTG	TGGATGCTCTCATCAGGACAG
IL-6	CTGCAAGAGACTTCCATCCAG	AGTGGTATAGACAGGTCTGTTGG
TNF-α	CAGGCGGTGCCTATGTCTC	CGATCACCCCGAAGTTCAGTAG
NF-κB	ATGGCAGACGATGATCCCTAC	CGGAATCGAAATCCCCTCTGTT
GAPDH	AGGTCGGTGTGAACGGATTTG	AGGTCGGTGTGAACGGATTTG

### Histological examination

After 14 days of monitoring, mice from each group were sacrificed. The upper portion of the left lungs was fixed in formalin and embedded in paraffin. Ultrathin sections (4-μm) were cut and stained with hematoxylin & eosin (HE). Lung histopathology was photographed under light microscopy (Olympus, Japan). Four readily identifiable pathological processes were graded semiquantitatively on a scale of 0 to 4 based on a similar scaling method (24): a score of 0 represented normal lung; 1 represented mild infiltrate; 2 was moderate infiltrate; 3 was severe infiltrate; and 4 denoted very severe changes. For each mouse, the score of inflammatory infiltrate in lungs was reached without controversy after reviewing the slides by two pathologists in a blinded manner.

### Statistical analysis

Data are reported as means±SD. A two-tailed Student's *t*-test was used to compare means of two groups and one-way and two-way analysis of variance (ANOVA) were used for comparisons of means of ≥3 groups, followed by Bonferroni post-hoc test. The survival analysis was performed by the Kaplan-Meier method and evaluated by means of log-rank (Mantel-Cox) test. Tests yielding 2-tailed values of P<0.05 were considered statistically significant. All experiments were repeated at least three times unless otherwise stated.

## Results

### Lapiferin attenuated H1N1-induced mortality and histopathology in lungs

Lapiferin was administered intraperitoneally 3 days post virus injection since mouse survival and body weight dropped dramatically on this time point (Figure 1A, D, and E). Neither control (PBS-injected) nor lapiferin-treated mice presented inflammatory infiltrate. However, the lungs in the H1N1-infected mice were full of inflammatory cells and showed alveolar consolidation. Interstitial edema and vascular congestion were observed in H1N1-infected lungs. In contrast, mice receiving lapiferin after H1N1 infection showed less severe inflammatory infiltrate. Interstitial edema was also alleviated by lapiferin (Figure 1B). Quantification of inflammatory infiltrate further showed that while H1N1 significantly promoted immune cells accumulation in lungs, co-treatment with lapiferin significantly attenuated the inflammatory infiltrate (Figure 1C). Moreover, while H1N1 caused significant mortality (40% died by the end of day 14), lapiferin significantly improved survival after its administration (Figure 1D).

Body weight increased in control and lapiferin-treated mice, however, H1N1-infected mice were weak and oligolectic and their body weight progressively declined. Administration of lapiferin increased daily activity (i.e., mice were more energetic and active to seek food) and thus alleviated body weight loss (Figure 1E). In addition, while lapiferin alone caused no effect on pulmonary edema or viral genesis in healthy mice, the H1N1 infection-induced pulmonary edema was significantly ameliorated by lapiferin co-treatments at 4 DPI as shown by the wet-to-dry ratios (Table 2). Viral load in lungs were also decreased by lapiferin co-treatments (Table 2). These data suggested that lapiferin attenuated H1N1-induced mortality and lung injuries.

**Table 2:** Wet-to-dry weight ratios and viral loads of lungs at 4 days post-infection.

Group	Wet/dry weight	Viral loads
Control	3.12±0.11	0.00±0.00
H1N1	7.41±0.62^a^	4.72±0.31^a^
Lapiferin	2.98±0.15	0.00±0.00
H1N1+Lapiferin	4.58±0.51^b^	4.21±0.12^b^
Ceruletide	3.69±0.71	0.00±0.00
H1N1+Ceruletide	8.94±0.61^b^	5.09±0.16^b^
H1N1+Ceruletide+Lapiferin	7.21±0.46^c,d^	4.87±0.32^c,d^

Data are reported as means±SD for n=5 per group. ^a^P<0.05 *vs* control; ^b^P<0.05 *vs* H1N1 group; ^c^P<0.05 *vs* H1N1+Lapiferin group; ^d^P<0.05 *vs* H1N1+ceruletide group (ANOVA).

### Lapiferin decreased expression of pro-inflammatory factors at both mRNA and protein levels in lungs

**Figure 2:**
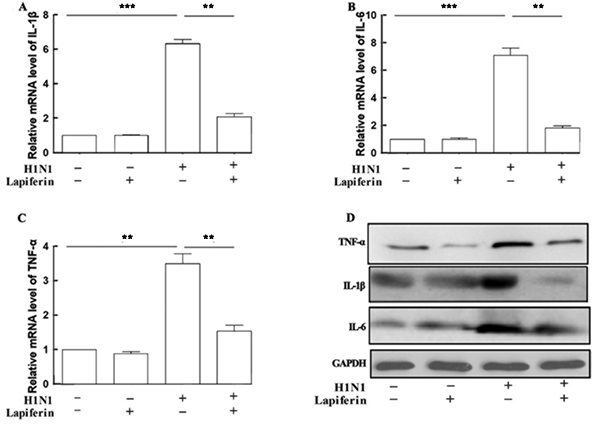
Lapiferin treatment decreased expression of pro-inflammatory factors at both mRNA and protein levels in lung. **A**, **B**, and **C**, qRT-PCR analysis of the mRNA levels of major pro-inflammatory factors (IL-1β, IL-6, and TNF-α) in the lung tissues from each group. **D**, Western blot analysis of the protein levels of IL-1β, IL-6, and TNF-α in the lung tissues of mice. Data are reported as means±SD. **P<0.01; ***P<0.001 as indicated (ANOVA).

Lapiferin treatment tended to decrease the mRNA levels of IL-1β, IL-6, and TNF-α compared with control mice, though this effect was not significantly different. Furthermore, H1N1 infection caused significant elevation of mRNA levels by up to 3-6-fold for IL-1β, IL-6, and TNF-α (Figure 2A-C), reinforcing the successful establishment of virus-induced pneumonia. More importantly, lapiferin administration to H1N1-infected mice significantly decreased the transcriptional levels of those pro-inflammatory cytokines in lung tissues (Figure 2A-C). Western blot analysis further validated that H1N1 infection caused consistent increases of IL-1β, IL-6, and TNF-α protein levels, whereas lapiferin administration blunted these H1N1-induced effects, though lapiferin seemed not to function in the absence of H1N1 infection (Figure 2D). These data suggested that lapiferin treatments decreased expression of pro-inflammatory factors at both mRNA and protein levels in lungs.

### Lapiferin decreased the levels of pro-inflammatory factors in lung homogenates

**Figure 3:**
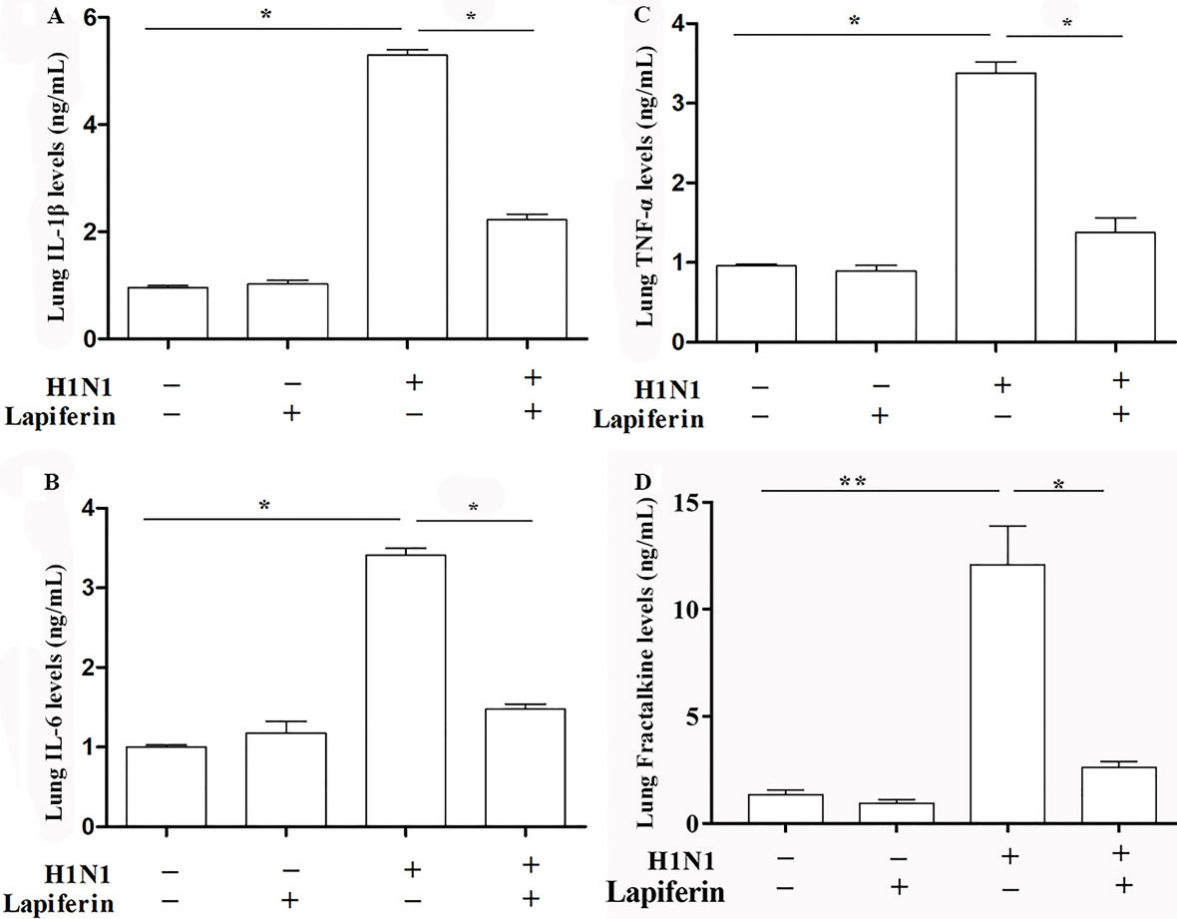
Lapiferin treatment decreased the serum levels of pro-inflammatory factors of H1N1 infected mice. **A**, **B**, **C**, and **D**, ELISA analysis of major pro-inflammatory factors (IL-1β, IL-6, and TNF-α) and fractalkine in the lung homogenates from all groups. Data are reported as means±SD. *P<0.05 and **P<0.01 as indicated (ANOVA).

The level of IL-1β was significantly increased by more than 5-fold in H1N1-treated mice compared to the control group (5.24±0.18 *vs* 1.05±0.02, P<0.05) (Figure 3A). Moreover, the co-treatment of H1N1 and lapiferin reversely decreased the level of IL-1β to the H1N1 group (5.24±0.18 *vs* 2.14±0.15, P<0.05). The level of IL-6 was enhanced by H1N1 infection (3.48±0.11 in H1N1 group *vs* 1.02±0.01 in control group, P<0.05, Figure 3B) but was blunted to the baseline level after lapiferin treatment (3.48±0.11 in H1N1 group *vs* 1.37±0.14 in lapiferin group, P<0.05, Figure 3B). Likewise, the TNF-α level was increased by approximately 3-fold after H1N1 infection (3.32±0.21 in H1N1 group *vs* 1.02±0.01 in control group, P<0.05, Figure 3C) and returned to a level comparable to the control after lapiferin treatment (3.32±0.21 in H1N1 group *vs* 1.31±0.11 in lapiferin group, P<0.05, Figure 3C). In addition, H1N1 infection significantly increased the level of Fractalkine by approximate 12-fold but lapiferin significantly dampened the H1N1-induced increases in Fractalkine in lung homogenates (Figure 3D). Together with Figure 2, these results illustrated that treatment with lapiferin rescued the inflammation caused by H1N1 infection.

### Lapiferin decreased inflammatory cell numbers in BAL fluid

**Figure 4:**
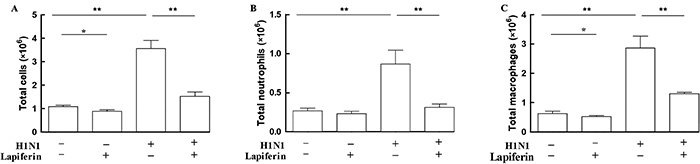
Lapiferin treatment decreased the number of inflammatory cells in bronchoalveolar fluid. The total inflammatory cell number (**A**), neutrophil number (**B**), and macrophage number (**C**) were counted in the bronchoalveolar fluid. Data are reported as means±SD. *P<0.05 and **P<0.01 as indicated (ANOVA).

Lapiferin alone caused significant inhibition of total inflammatory cells in the BAL fluid, which contrasted with H1N1 that promoted the release of total inflammatory cells into BAL fluid by up to 3.5-folds. Lapiferin administration to H1N1-infected mice significantly decreased the number of inflammatory cells in the BAL fluid (Figure 4A). Accordingly, neutrophils in the BAL fluid were significantly increased by H1N1 infection (220% increase), while this effect was blunted by lapiferin, which decreased the neutrophil numbers to a level comparable to control (62.5% inhibition, Figure 4B). Similarly, macrophages were the major inflammatory cell type in the BAL fluid. The number of macrophages in the BAL fluid increased by nearly 3-fold after H1N1 infection, but significantly decreased after lapiferin administration (60.8% inhibition, Figure 4C). These observations suggested that lapiferin treatments decreased the number of inflammatory cell in the BAL fluid.

### Lapiferin suppressed NF-κB signaling

**Figure 5:**
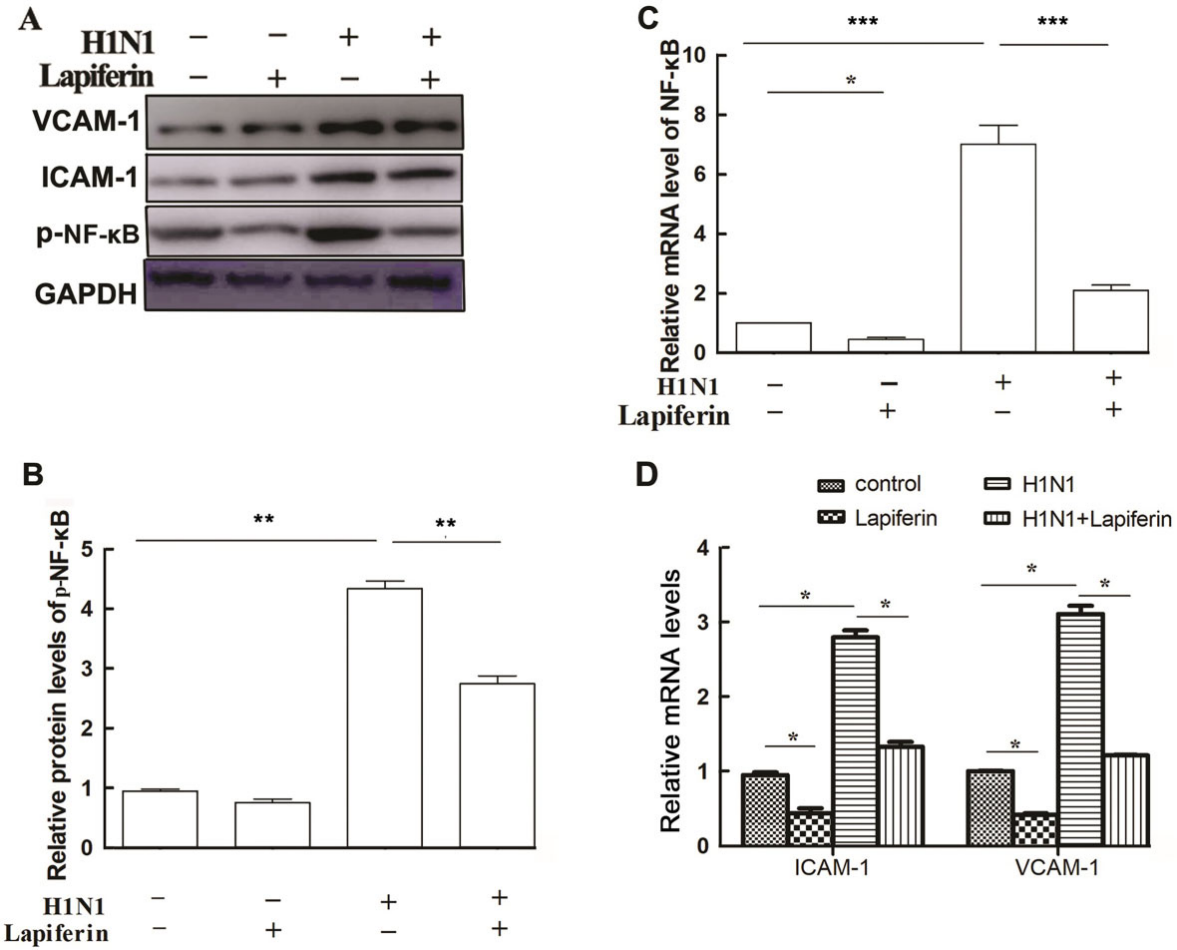
Lapiferin suppressed NF-κB signaling. **A** and **B**, Western blot analysis and quantitative analysis of NF-κB (p65) in the lung homogenate from each group of mice. **C**, qRT-PCR analysis of NF-κB in the lung homogenate from each group of mice. **D**, mRNA levels of *ICAM-1* and *VCAM-1* in mouse lung tissues. Data are reported as means±SD. *P<0.05; **P<0.01; ***P<0.001 as indicated (ANOVA).

H1N1 infection caused significant increases of the active form of NF-κB (phosphorylated NF-κB, Figure 5A), which was consistent with previous observations (20,25). Administration of lapiferin to H1N1-infected mice also ameliorated the NF-κB p65 protein level in the lung homogenates (Figure 5B). Of note, the protein levels of inflammatory molecules ICAM-1 and VCAM-1 were also decreased by lapiferin and increased by H1N1 infection (Figure 5A). As a reflection of NF-κB transcriptional activity, the mRNA level of NF-κB p65 was also observed to be significantly decreased by lapiferin administration in mice (Figure 5C). Furthermore, the mRNA levels of ICAM-1 and VCAM-1 showed a similar tendency with that of NF-κB (Figure 5D). These data indicated that lapiferin treatments suppressed NF-κB signaling.

### Activation of NF-κB signaling blocked lapiferin-mediated anti-inflammatory effects *in vitro* and *in vivo*

**Figure 6:**
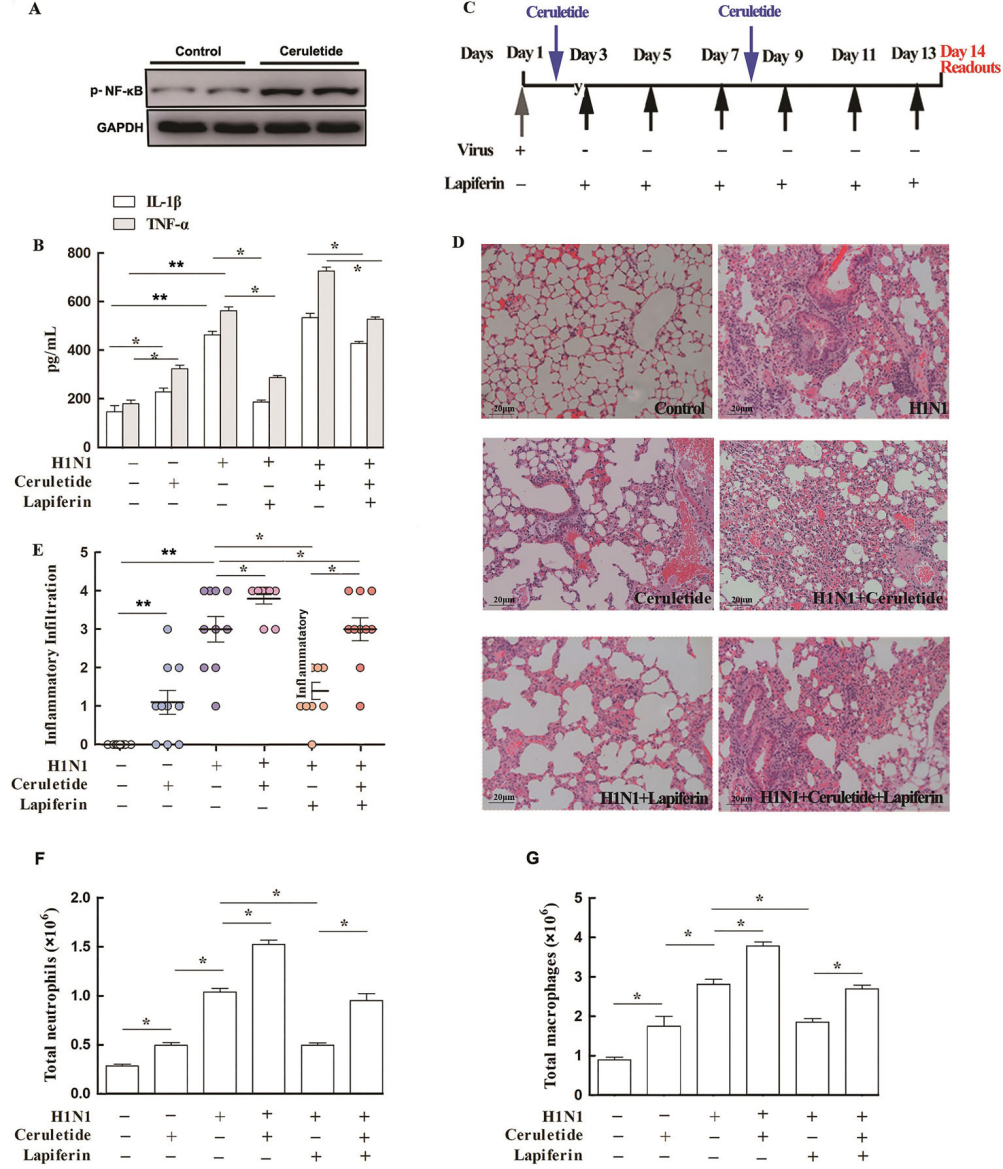
Activation of NF-κB signaling blocked lapiferin-mediated anti-inflammatory effects *in vitro* and *in vivo*. **A**, Western blot assays validated the activation of NF-κB signaling by its specific activator ceruletide. **B**, ELISA detection of pro-inflammatory cytokines IL-1β and TNF-α in the supernatant of cultured human bronchial epithelial cells. **C**, Experimental protocol for the *in vivo* studies. **D**, Representative histology images after 14 days (magnification: 400×, scale bar: 20 μm). **E**, Semiquantitative analysis of lung inflammatory infiltrate. **F**, Total neutrophils levels and (**G**) total macrophage levels are shown. Data are reported as means±SD. *P<0.05; **P<0.01 as indicated (ANOVA).

In view of the alteration of NF-κB signaling, we then employed a specific activator of NF-κB signaling, ceruletide, to re-express NF-κB (Figure 6A) and examined the changes in the effect of lapiferin. In cultured human bronchial epithelial cells, lapiferin significantly decreased the H1N1-induced release of IL-1β and TNF-α into cell supernatants. However, after activation of NF-κB signaling by its specific activator ceruletide, the levels of IL-1β and TNF-α in cell supernatants were unchanged compared to the H1N1 infection group (Figure 6B). In the *in vivo* assays, after H1N1 infection, ceruletide was administered prior to lapiferin injection (on day 2 and day 8, respectively) to recover NF-κB signaling in advance (Figure 6C). As shown in Figure 6D and E, H1N1 caused severe inflammation in lung tissues. Lapiferin significantly blunted H1N1-induced inflammation. However, when NF-κB signaling was re-activated by ceruletide in advance, lapiferin failed to attenuate inflammatory infiltrate in the lung tissues. The total neutrophils and total macrophage levels were consistent with the above observations (Figure 6F and G). Pre-activation of NF-κB signaling also blunted the lapiferin-mediated protection from H1N1-induced pulmonary edema and viral loading in lungs (Table 2). These data suggested that lapiferin protected against virus-induced inflammation *via* negatively regulating NF-κB signaling.

## Discussion

The typical manifestations of H1N1 infection include fever, cough, sore throat, chills, headache, rhinorrhea, shortness of breath, myalgias, arthralgias, fatigue, vomiting, and diarrhea. Though most patients present with mild illness, some have a severe course that may result in respiratory failure and death, especially young patients (25). The current antiviral agents are restricted from clinical application due to their low efficacy or delayed response (8). Thus, novel effective therapeutic strategies against H1N1 infection should be investigated.

Lapiferin structure and configuration have been established based on chemical transformations and analysis of spectral characteristics (26). After establishment of a mouse pneumonia model, we observed that lapiferin administration significantly alleviated lung injury and improved survival (27). High levels of pro-inflammatory cytokines such as IL-1β, IL-6, and TNF-α play an essential role in the inflammatory response, and strategies to inhibit their functions confer therapeutic potential (28). After lapiferin administration, the expression levels of IL-1β, IL-6, and TNF-α were significantly decreased at both mRNA and protein levels, confirming our histological observation that lapiferin alleviated lung histopathology in mice. In addition, we observed that the immune cells, including macrophages and neutrophils, underwent a steep increase in the BAL fluid with H1N1 infection, but significantly decreased in numbers after lapiferin administration. With H1N1 infection, human macrophages and neutrophils produced significantly higher levels of pro-inflammatory cytokines and chemokines, such as TNF-α, IL-1β, IL-6, and fractalkine, compared to that with no virus infection (20). These cytokines recruit and/or activate a variety of immune cells and immunocompetent cells, such as macrophages, neutrophils, and epithelial cells. Upon activation, these immune cells further secrete a range of potent pro-inflammatory cytokines such as IL-6 and TNFα (29). The mutual activation between cytokines and immune cells eventually leads to further endothelial cell activation, amplified inflammation, and impaired epithelial barrier function by potentially increasing the susceptibility to viral/bacterial infection and subsequent exacerbations (30). In view of previous reports and our observations, the lapiferin-mediated decrease of immune cells in the BAL fluid and suppression of pro-inflammatory factors and chemokine in lung tissues reinforced that lapiferin exerted potent anti-inflammatory activity against H1N1 infection in lungs.

In addition, lapiferin administration significantly suppressed NF-κB expression after H1N1 challenge. Using the specific activator ceruletide to re-express NF-κB, the lapiferin-mediated anti-inflammatory activity was no longer observed *in vitro* and *in vivo*. These data were conclusive that lapiferin protected against H1N1-induced pneumonia *via* suppressing NF-κB signaling. However, the explicit mechanisms of how lapiferin negatively regulated NF-κB remain to be elucidated.

One interesting point was that H1N1 exacerbated the number of macrophages in BAL of mice as observed in the current study. Macrophages are the first line of defense against viral infection, including influenza virus infection (31). In the healthy lung, at least two macrophage populations exist, namely, alveolar macrophages and interstitial macrophages (32). Alveolar macrophages are classified as M1 and M2 macrophages according to their inflammatory state (33). M1 macrophages produce proinflammatory cytokines, thus contributing to host defense against pathogens and tissue injury; M2 macrophages produce anti-inflammatory cytokines, thus promoting tissue repair (32,33). The switch of M1/M2 phenotypes is referred to as macrophage polarization, which could be validated by multiple phenotypic markers, cytokines, and growth factors, such as nitric oxide synthase, TNF-α, and IL-10 (34). Macrophage polarization can occur at any point in multiple inflammatory processes (31). The phenomenon of H1N1-induced excessive accumulation of macrophages in BAL mandates further investigation on whether macrophage polarization was critically involved in the H1N1-induced lung inflammatory injuries.

The identification of lapiferin as a potential anti-inflammatory agent under virus challenge is of great biological significance. First, the anti-inflammatory activity of lapiferin had not been reported and our report represented the first one to uncover the novel function. Second, lapiferin protected against H1N1-induced pneumonia via suppressing NF-κB signaling. Modulation of NF-κB signaling has been shown to have therapeutic potentials in autoimmunity (30). Our data might suggest that specific NF-κB inhibitors may synergize with lapiferin to impose its anti-inflammatory property.

In summary, the present study identified lapiferin as a potent anti-inflammatory agent under virus challenge. Our data might pave the way to novel insights into the development of therapeutic agents against pandemic virus infections.

## Supplementary Material

Click here to view [pdf].
